# Association of Cumulative Colorectal Surgery Hospital Costs, Readmissions, and Emergency Department/Observation Stays with Insurance Type

**DOI:** 10.1007/s11605-022-05576-7

**Published:** 2023-01-23

**Authors:** Michael A. Jacobs, Jasmine C. Tetley, Jeongsoo Kim, Susanne Schmidt, Bradley B. Brimhall, Virginia Mika, Chen-Pin Wang, Laura S. Manuel, Paul Damien, Paula K. Shireman

**Affiliations:** 1 Department of Surgery, University of Texas Health San Antonio, San Antonio, TX USA; 2 Department of Population Health Sciences, University of Texas Health San Antonio, San Antonio, TX USA; 3 Department of Pathology and Laboratory Medicine, University of Texas Health San Antonio, San Antonio, TX USA; 4 University Health, San Antonio, TX USA; 5 Business Intelligence and Data Analytics, University of Texas Health Physicians, University of Texas Health San Antonio, San Antonio, TX USA; 6 Department of Information, Risk, and Operations Management, School of Business, University of Texas, Red McCombs, Austin, TX USA; 7 Departments of Primary Care & Rural Medicine and Medical Physiology, School of Medicine, Texas A&M Health, Bryan, TX USA

**Keywords:** Urgent surgery, Variable costs, Vulnerable populations

## Abstract

**Background/Purpose:**

Medicare’s Hospital Readmission Reduction Program disproportionately penalizes safety-net hospitals (SNH) caring for vulnerable populations. This study assessed the association of insurance type with 30-day emergency department visits/observation stays (EDOS), readmissions, and cumulative costs in colorectal surgery patients.

**Methods:**

Retrospective inpatient cohort study using the National Surgical Quality Improvement Program (2013–2019) with cost data in a SNH. The odds of EDOS and readmissions and cumulative variable (index hospitalization and all 30-day EDOS and readmissions) costs were modeled adjusting for frailty, case status, presence of a stoma, and open versus laparoscopic surgery.

**Results:**

The cohort had 245 private, 195 Medicare, and 590 Medicaid/uninsured cases, with a mean age 55.0 years (SD = 13.3) and 52.9% of the cases were performed on male patients. Most cases were open surgeries (58.7%). Complication rates were 41.8%, EDOS 12.0%, and readmissions 20.1%. Medicaid/uninsured had increased odds of urgent/emergent surgeries (aOR = 2.15, CI = 1.56–2.98, *p* < 0.001) and complications (aOR = 1.43, CI = 1.02–2.03, *p* = 0.042) versus private patients. Medicaid/uninsured versus private patients had higher EDOS (16.6% versus 4.1%) and readmissions (22.9% versus 14.3%) rates and higher odds of EDOS (aOR = 4.81, CI = 2.57–10.06, *p* < 0.001), and readmissions (aOR = 1.62, CI = 1.07–2.50, *p* = 0.025), while Medicare patients had similar odds versus private. Cumulative variable cost %change was increased for Medicare and Medicaid/uninsured, but Medicaid/uninsured was similar to private after adjusting for urgent/emergent cases.

**Conclusions:**

Increased urgent/emergent cases in Medicaid/uninsured populations drive increased complications odds and higher costs compared to private patients, suggesting lack of access to outpatient care. SNH care for higher cost populations, receive lower reimbursements, and are penalized by value-based programs. Increasing healthcare access for Medicaid/uninsured patients could reduce urgent/emergent surgeries, resulting in fewer complications, EDOS/readmissions, and costs.

**Supplementary Information:**

The online version contains supplementary material available at 10.1007/s11605-022-05576-7.

## Introduction

Colorectal surgeries (CRS) are among the most commonly performed procedures with high rates of complications,^1^ emergency department visits/observation stays (EDOS),^2^ and readmissions.^2^ CRS are resource intensive, and readmissions can more than double median hospital costs.^3,4^ However, readmissions may not reflect quality of care as a quality metric.^3^ For example, safety-net hospitals (SNH) have higher rates of complications and readmissions,^5^ but care for higher proportions of vulnerable patients who have increased rates of poor outcomes.^5^ Low socioeconomic status (SES) populations are associated with greater risk of morbidity,^6^ mortality,^5^ and poor health status.^6^ Current risk adjustment models do not include frailty^7–9^ and social risk factors^10–12^ which significantly impact patient outcomes.^11–15^ With the higher costs of care for low-SES populations, in addition to lower reimbursements, SNH are further penalized by current value-based programs.^16,17^

The Hospital Readmissions Reduction Program (HRRP) is a Medicare value-based program used as a quality measure and to decrease healthcare spending. HRRP reduces payments to hospitals with higher 30-day readmissions than predicted by risk-adjustment models.^18^ The payment reductions have disproportionately penalized SNHs.^19–24^ Stratifying hospitals into five groups based upon percent of dual enrollment Medicare–Medicaid patients reduced but did not eliminate SNH penalties.^25^ The typical SNH still receives a 0.28% reduction across all Medicare payments and 91.6% of low-socioeconomic status/high-burden hospital experience penalties.^25^ Readmission penalties in 2019 impacted 75% of all hospitals.^25^

Our primary clinical objective was to assess the association of insurance type with 30-day EDOS and readmissions in an academic SNH serving a diverse population. Our cost objectives were to assess the association of insurance type with (1) the variable costs of the index hospitalization for patients without and with EDOS or readmissions, (2) the costs of the initial EDOS or readmission, (3) the cumulative costs of the index hospitalization and all 30-day EDOS and readmissions, and (4) return costs (sum of all 30-day EDOS and readmissions).

## Materials and Methods

### Study Population and Data

This study used a retrospective cohort of inpatients undergoing CRS using data in the 2013–2019 American College of Surgeons National Surgical Quality Improvement Program (NSQIP) at an academic medical center and SNH following STROBE Reporting Guidelines. NSQIP started at our hospital in April 2013 with random selection of colorectal surgeries. In mid-2016 all eligible colorectal surgeries were included in NSQIP. NSQIP registry provides standardized definitions of preoperative risk factors and complications.^26–28^ Patient self-reported race and ethnicity were derived from NSQIP variables and electronic health records (EHR). Dates of death were obtained from NSQIP and supplemented by EHR and the Social Security Death Master File.^29^ The Institutional Review Board (IRB) of the University of Texas Health San Antonio approved this study.

### Estimating Patient Frailty/Premorbid Conditions

Frailty was measured using the recalibrated Risk Analysis Index (RAI)^7^ using preoperative variables from NSQIP.^30,31^ RAI has been validated using NSQIP and Veterans Affairs Surgical Quality Improvement Program datasets.^7,31,32^ RAI exhibits collinearity^33^ with the Charlson Comorbidity Index. RAI is used as a single-variable estimate of patient-level variability that overcomes barriers to model fit encountered by less parsimonious models and has been used to risk adjust for medical comorbid conditions in multiple studies.^30,34–38^ RAI scores were grouped into robust (≤ 20), normal (21–29), frail (30–39), and very frail (≥ 40), as previously described.^30,34,37,38^

### Procedure Identification, Stoma, Case Status, and 30-Day Complications

CRS were identified and categorized as open or laparoscopic surgeries using NSQIP principal Current Procedural Terminology (CPT) codes (Supplementary Table 1). All CPT codes used for billing during the index hospitalization were assessed; patients with any CPT code specifying operative placement of a stoma or associated with the presence of a stoma (e.g., endoscopy performed through a stoma) were classified as having stoma regardless of the principal CPT code. Chart review was performed on cases with CPT codes with optional stoma placement to determine stoma status (Supplementary Table 1**).**

Case status was determined from NSQIP variables with urgent cases being defined as “no” responses to elective and emergency variables.^39^ Any complication was defined using the 20 NSQIP variables defining postoperative complications (Supplementary Table 2).

### 30-Day EDOS and Readmissions

NSQIP only tracks patients for 30 days after surgery and contains 30-day readmission variables from the date of surgery. We merged NSQIP data with EHR to determine readmissions and EDOS within 30 days of discharge from the index procedure’s hospitalization, to be consistent with the HRRP definition of 30-day readmissions.

### Insurance Type and Cost Data

The identified, local NSQIP data were merged with EHR and managerial accounting data to determine insurance type and cost of the index hospitalization, readmissions, and EDOS. Insurance type was categorized based upon billing data for the encounter supplemented by EHR data and defined as (1) private insurance including workers’ compensation, (2) Medicare, and (3) Medicaid/uninsured including Medicaid, dual enrollment in Medicare/Medicaid, Charity Care, self-pay, or county indigent care programs (Supplementary Table 3). Patients enrolled in both Medicare and Medicaid were allocated only to the Medicaid/uninsured group. “Other” included encounters billed to the Veterans Administration, Department of Corrections, or self-pay with > 1% of charges collected and were excluded.

Our hospital used EPSi for internal cost accounting. EPSi provides an array of accounting functions including real-time costing, budgeting, productivity measurement, and decision support. We defined variable costs as costs related directly to patient care occurring during the encounter, such as supplies and salaries, and include direct variable costs that vary directly with the quantity of resources provided for patient care. Variable costs were derived using direct measurements from a bottom-up approach, rather than calculated estimates derived from charges. Fixed costs include money used on facilities such as insurance for buildings and equipment and do not vary with the number of patients served. Fixed costs represent a substantial portion of a hospital’s budget^40,41^ and cannot be reduced in the short term.^42^ We used variable costs, as fixed costs are not directly related to patient care and vary between hospitals.^43^ Hospital fixed costs, outpatient, and professional fees were not included as the focus of this study was on variable costs to the hospital for an inpatient surgical procedure and subsequent EDOS and readmissions.^44^ The natural logarithm of variable costs was used, as previously described,^45^ after adjusting costs to 2019 dollars using the Personal Health Care Index.^46^

### Exclusions

Cases were excluded due to (1) perineal or sacral procedures, (2) other insurance type, and (3) missing cost data for index hospitalization or readmission/EDOS costs, including readmissions to outside hospitals.

**Fig. 1:**
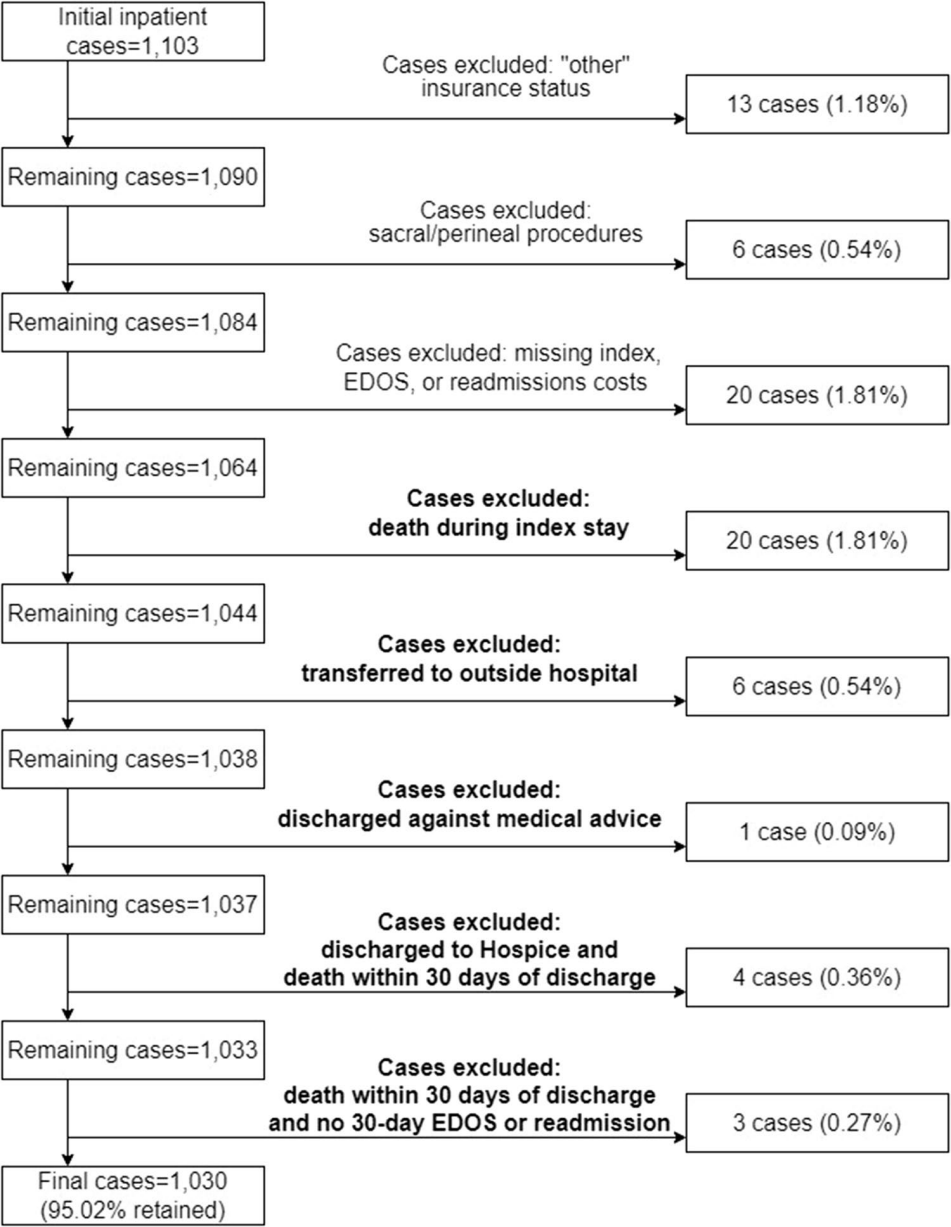
Cohort flow diagram. National Surgery Quality Improvement Program inpatient cases from 2013 to 2019. Cases were excluded for perineal or transsacral only procedures, “other” insurance status, and missing cost data. “Other” insurance status was defined as included encounters billed to the Veterans Administration, Department of Corrections, or self-pay with > 1% of charges collected. Cases were excluded for missing cost data and for patient mortality resulting in no or reduced chances of subsequent Emergency Department Visits/Observation Stays (EDOS) and readmissions leaving a final study cohort of 1030 cases. A sensitivity analysis was performed to add 34 previously excluded cases; the cases added for the sensitivity analysis are marked with bold text

Patient mortality resulting in no or reduced chances of subsequent EDOS and readmissions were excluded, as previously described.^20^ Cases were excluded due to (1) death during the index hospitalization, (2) discharge to another acute care hospital, (3) discharge against medical advice, (4) death within 30 days of discharge when discharged to Hospice or Home on Hospice, and (5) death within 30 days of discharge without a 30-day EDOS or readmission. A sensitivity analysis was performed adding groups 1–5 (34 cases) to ensure clinical outcomes were robust to cohort selection (Fig. 1).

### Study Outcomes

Our main clinical analyses were the association of insurance type with EDOS and readmissions. Sub-analyses assessed the association of insurance type with EDOS and readmission risk factors of urgent/emergent cases,^47^ open abdominal procedures,^3^ presence of a stoma,^3,47^ and postoperative complications.^47^

Our cost analyses included the association of insurance type with (1) index hospitalization costs for patients without and with an EDOS or readmission, (2) costs of initial EDOS and readmission, (3) cumulative costs of the index hospitalization and subsequent 30-day EDOS and readmissions, and (4) return costs (sum of all 30-day EDOS and readmissions).

### Statistical Analysis

Categorical data was summarized using count and percentage, with continuous data using mean and standard deviation (SD) or medians with quartiles. Chi-square tests and *F*-tests tested differences between groups for categorical and continuous variables, except length of stay (LOS) and variable costs for (1) the index hospitalization, (2) EDOS, (3) readmissions, (4) cumulative costs, and (5) return costs which used Kruskal–Wallis tests. Logistic regression analyses were performed for case status and complications adjusting for a combination of RAI, open versus laparoscopic procedure, case status, and insurance type. Natural logarithms normalized the skewed LOS and variable costs reducing the impact of outliers, as previously described.^45,48^ Percent change/relative difference was calculated using the exponential function; %change = (e^Estimate coefficients^ − 1)*100. Analyses were performed using R 4.1.0 (2021–05-18).

## Results

### Population Demographics

Our cohort consisted of 1030 cases of colorectal procedures (Fig. 1). Most cases (Table 1) were performed on male (52.9%) and White patients (90.1%), with a majority identifying as Hispanic (64.6%). Cases were performed on Medicaid/uninsured insurance patients (57.3%), private (23.8%), and Medicare (18.9%). Most patients were robust (61.7%) or normal (27.1%) based on RAI scores. Only 9.2% and 1.9% of cases involved frail and very frail patients, respectively, with Medicare patients having higher frailty scores.

**Table 1:** Patient characteristics and clinical outcomes by insurance type

	Overall	Private	Medicare	Medicaid/uninsured	*p* value
Number (%)^a^	1030	245 (23.8)	195 (18.9)	590 (57.3)	
Age mean (SD)	55.0 (13.3)	51.2 (11.2)	68.0 (10.0)	52.2 (12.5)	**< 0.001**
Sex					0.854
Female	485 (47.1)	119 (48.6)	92 (47.2)	274 (46.4)	
Male	545 (52.9)	126 (51.4)	103 (52.8)	316 (53.6)	
Race					0.430
White	928 (90.1)	217 (88.6)	182 (93.3)	529 (89.7)	
Black	72 (7.0)	19 (7.8)	8 (4.1)	45 (7.6)	
Other^b^	30 (2.9)	9 (3.7)	5 (2.6)	16 (2.7)	
Hispanic ethnicity	665 (64.6)	117 (47.8)	108 (55.4)	440 (74.6)	**< 0.001**
RAI (frailty)					**< 0.001**
Robust (≤ 20)	636 (61.7)	191 (78.0)	43 (22.1)	402 (68.1)	
Normal (21–29)	279 (27.1)	42 (17.1)	104 (53.3)	133 (22.5)	
Frail (30–39)	95 (9.2)	11 (4.5)	40 (20.5)	44 (7.5)	
Very frail (≥ 40)	20 (1.9)	1 (0.4)	8 (4.1)	11 (1.9)	
Case status					**< 0.001**
Elective	608 (59.0)	175 (71.4)	124 (63.6)	309 (52.4)	
Urgent	323 (31.4)	56 (22.9)	45 (23.1)	222 (37.6)	
Emergent	99 (9.6)	14 (5.7)	26 (13.3)	59 (10.0)	
Laparoscopic	425 (41.3)	115 (46.9)	86 (44.1)	224 (38.0)	**0.038**
Open abdomen	605 (58.7)	130 (53.1)	109 (55.9)	366 (62.0)	**0.038**
Stoma status	448 (43.5)	88 (35.9)	92 (47.2)	268 (45.4)	**0.021**
Any complication	431 (41.8)	78 (31.8)	92 (47.2)	261 (44.2)	**0.001**
Length of stay mean (SD)	10.1 (10.9)	8.1 (10.2)	10.7 (10.2)	10.7 (11.3)	**< 0.001**^c^
Median (Q1–Q3)	7 (4–12)	5 (4–8)	7 (4–14)	7 (5–13)	
30-day EDOS^d^	124 (12.0)	10 (4.1)	16 (8.2)	98 (16.6)	**< 0.001**
30-day readmissions^d^	207 (20.1)	35 (14.3)	37 (19.0)	135 (22.9)	**0.017**

*EDOS*, emergency department visits/observation stays; *Q*, quartile; *RAI*, risk analysis index; *SD*, standard deviation

^a^Percent calculations by row, the rest of the percent calculations were by column

^b^Other includes 20 Asian, 1 American Indian or Alaska Native, and 9 multi-racial

^c^*p* value based on a Kruskal–Wallis test due to high skewedness of length of stay

^d^30-day EDOS and readmission defined as 30 days from date of discharge from index hospitalization

Readmissions and EDOS were evaluated independently; membership in one group does not exclude a case from membership in the other. P values in bold indicate significance at the *p* < .05 level

#### Clinical Analysis Outcomes

### Increased Urgent/Emergent Cases in Medicare and Medicaid/Uninsured Patients with Similar Odds of Having a Stoma and Open Abdominal Procedure

Urgent/emergent surgeries were higher in Medicaid/uninsured (47.6%) and Medicare (36.4%) patients versus private (28.6%, Table 1). Medicaid/uninsured (aOR = 2.15, CI = 1.56–2.98, *p* < 0.001), but not Medicare patients, had higher odds of an urgent/emergent surgeries versus private (Table 2). Increased odds of having a stoma or undergoing an open abdominal procedure were associated with frail and very frail patients (Table 2 M1 and M2). Adjusting for urgent/emergent cases (Table 2 M2) increased the odds of having a stoma (aOR = 2.53, CI = 1.95–3.31, *p* < 0.001) and undergoing an open abdominal procedure (aOR = 2.80, CI = 2.13–3.70, *p* < 0.001). Medicaid/uninsured (aOR = 1.37, CI = 1.01–1.86, *p* = 0.043) patients demonstrated higher odds of undergoing open abdominal surgery versus private (Table 2 M1) but were similar to private after adjusting for urgent/emergent cases (Table 2 M2).

**Table 2:** Urgent/emergent cases, stoma, and open abdominal procedures adjusted odds ratios

	Urgent/emergent cases			Stoma M1		Open abdomen M1			
Predictors	aOR	CI	*p* value	aOR	CI	*p* value	aOR	CI	*p* value
RAI (Ref = normal 21–29)									
Robust (≤ 20)	0.66	0.48–0.90	**0.009**	0.64	0.47–0.86	**0.004**	0.83	0.61–1.13	0.231
Frail (30–39)	1.60	0.99–2.58	0.054	3.12	1.89–5.28	**< 0.001**	2.35	1.40–4.05	**0.002**
Very frail (≥ 40)	2.95	1.13–8.64	**0.034**	6.51	2.12–28.40	**0.003**	6.60	1.85–42.15	**0.013**
Insurance (Ref = private)									
Medicare	1.01	0.65–1.56	0.973	0.98	0.64–1.50	0.919	0.84	0.55–1.27	0.412
Medicaid/uninsured	2.15	1.56–2.98	**< 0.001**	1.36	1.00–1.87	0.054	1.37	1.01–1.86	**0.043**
					Stoma M2		Open abdomen M2		
Predictors				aOR	CI	*p* value	aOR	CI	*p* value
RAI (Ref = normal 21–29)									
Robust (≤ 20)				0.68	0.50–0.94	**0.018**	0.91	0.66–1.25	0.564
Frail (30–39)				2.96	1.77–5.05	**< 0.001**	2.17	1.28–3.80	**0.005**
Very frail (≥ 40)				5.51	1.76–24.31	**0.008**	5.39	1.47–34.81	**0.028**
Insurance (Ref = private)									
Medicare				0.99	0.64–1.53	0.948	0.84	0.55–1.29	0.432
Medicaid/uninsured				1.16	0.84–1.61	0.357	1.16	0.85–1.59	0.352
Urgent/emergent (Ref = elective)				2.53	1.95–3.31	**< 0.001**	2.80	2.13–3.70	**< 0.001**

*aOR*, adjusted odds ratio; *CI*, 95% confidence interval; *EDOS*, emergency department visits/observation stays; *M*, models 1 and 2; *RAI*, risk analysis index; *Ref*, reference value. P values in bold indicate significance at the *p* < .05 level

### Increased Complications in Medicare and Medicaid/Uninsured Patients

Higher odds of complications (Table 3 M1) occurred with open abdominal cases (aOR = 2.64, CI = 1.96–3.57, *p* < 0.001) and having a stoma (aOR = 3.39, CI = 2.55–4.53, *p* < 0.001). Complications were higher in Medicare (47.2%), followed by Medicaid/uninsured (44.2%) compared to private (31.8%, Table 1). Medicaid/uninsured (aOR = 1.43, CI = 1.02–2.03, *p* = 0.042) patients had higher odds of complications versus private (Table 3 M1). However, subgroup analysis for patients with a stoma showed similar odds of complications between all three insurance groups (Table 3 M2).

### Increased 30-Day EDOS and Readmissions Among Medicaid/Uninsured Patients

Medicaid/uninsured patients had the highest rates of EDOS (16.6%, Table 1) and increased odds of EDOS (aOR = 4.81, CI = 2.57–10.06, *p* < 0.001) in all cases versus private after adjusting for frailty, open abdomen vs. laparoscopic surgery, urgent/emergent cases, and stoma (Table 3 M1). In the stoma subgroup, Medicaid/uninsured patients (aOR = 3.94, CI = 1.72–10.70, *p* = 0.003) had higher odds of EDOS (Table 3 M2). Medicaid/uninsured patients in the all cases group continued to have higher odds of EDOS (aOR = 4.66, CI = 2.47–9.75, *p* < 0.001) even after adjusting for complications (Table 3 M3).

Odds of readmission (Table 3 M1) were increased with open abdominal surgeries (aOR = 1.73, CI = 1.20–2.51, *p* = 0.004) and having a stoma (aOR = 2.83, CI = 2.00–4.03, *p* < 0.001). Medicaid/uninsured patients demonstrated the highest rates of readmissions (22.9%; Table 1) and increased odds of readmissions (aOR = 1.62, CI = 1.07–2.50, *p* = 0.025) in all cases versus private after adjusting for frailty, open abdomen vs. laparoscopic surgery, urgent/emergent cases, and stoma (Table 3 M1). However, readmission odds in Medicaid/uninsured versus private were similar in the stoma subgroup (Table 3 M2) and also in the all cases group after adjusting for complications (Table 3 M3).

### Sensitivity Analyses of Clinical Outcomes

Sensitivity analyses added 34 cases initially excluded with no or reduced chances of subsequent EDOS and readmissions. The expanded cohort was assessed using the clinical outcome variables present in Tables 2 and 3. Results were similar between the original and expanded sensitivity analysis cohorts (Supplementary Fig. 1).

**Table 3:** Any complication, EDOS, and readmission adjusted odds ratios adjusted for frailty, open abdominal procedures, urgent/emergent cases, insurance type, and stoma status

	Any complication			30-day EDOS			30-day readmission		
Predictors	aOR	CI	*p* value	aOR	CI	*p* value	aOR	CI	*p* value
M1: all cases (*N* = 1030)									
RAI (Ref = normal 21–29)									
Robust (≤ 20)	0.90	0.65–1.27	0.558	1.13	0.71–1.84	0.626	1.10	0.74–1.64	0.645
Frail (30–39)	1.31	0.78–2.20	0.315	1.08	0.50–2.20	0.839	1.15	0.65–2.02	0.628
Very frail (≥ 40)	2.49	0.85–9.13	0.122	1.19	0.26–3.94	0.796	2.83	1.08–7.45	**0.032**
Open abdomen (Ref = laparoscopic)	2.64	1.96–3.57	**< 0.001**	1.13	0.74–1.75	0.564	1.73	1.20–2.51	**0.004**
Urgent/emergent (Ref = elective)	1.08	0.81–1.45	0.583	0.68	0.45–1.02	.066	0.79	0.57–1.11	0.178
Insurance (Ref = private)									
Medicare	1.57	0.99–2.49	0.057	2.16	0.94–5.20	0.076	1.22	0.70–2.14	0.481
Medicaid/uninsured	1.43	1.02–2.03	**0.042**	4.81	2.57–10.06	**< 0.001**	1.62	1.07–2.50	**0.025**
Stoma (Ref = none)	3.39	2.55–4.53	**< 0.001**	1.62	1.06–2.46	**0.025**	2.83	2.00–4.03	**< 0.001**
M2: stoma subgroup (*N* = 448)									
RAI (Ref = normal 21–29)									
Robust (≤ 20)	0.93	0.58–1.50	0.769	1.19	0.62–2.37	0.605	1.09	0.66–1.81	0.745
Frail (30–39)	1.50	0.79–2.93	0.219	1.23	0.50–2.92	0.642	1.24	0.64–2.36	0.517
Very frail (≥ 40)	2.58	0.77–11.75	0.159	0.94	0.14–3.89	0.937	2.99	1.05–8.68	**0.039**
Open abdomen (Ref = laparoscopic)	2.77	1.75–4.39	**< 0.001**	0.87	0.47–1.65	0.651	1.46	0.88–2.46	0.149
Urgent/emergent (Ref = elective)	1.14	0.76–1.70	0.535	0.45	0.26–0.78	**0.004**	0.67	0.44–1.01	0.055
Insurance (Ref = private)									
Medicare	1.20	0.62–2.34	0.589	1.87	0.61–6.11	0.278	0.96	0.47–1.95	0.910
Medicaid/uninsured	1.21	0.72–2.01	0.467	3.94	1.72–10.70	**0.003**	1.34	0.79–2.35	0.288
M3: all cases (*N* = 1030) adjusted for any complication									
RAI (Ref = normal 21–29)									
Robust (≤ 20)				1.14	0.71–1.86	0.595	1.13	0.76–1.71	0.549
Frail (30–39)				1.05	0.49–2.16	0.890	1.08	0.59–1.94	0.801
Very frail (≥ 40)				1.02	0.22–3.43	0.974	2.35	0.86–6.49	0.093
Open abdomen (Ref = laparoscopic)				0.96	0.62–1.49	0.843	1.26	0.85–1.87	0.251
Urgent/emergent (Ref = elective)				0.66	0.43–0.99	**0.047**	0.75	0.53–1.07	0.113
Insurance (Ref = private)									
Medicare				2.01	0.87–4.84	0.107	1.08	0.61–1.92	0.791
Medicaid/uninsured				4.66	2.47–9.75	**< 0.001**	1.51	0.98–2.38	0.066
Stoma (Ref = none)				1.29	0.84–2.01	0.248	1.97	1.36–2.86	**< 0.001**
Any complication (Ref = none)				2.31	1.51–3.58	**< 0.001**	4.78	3.30–7.03	**< 0.001**

*aOR*, adjusted odds ratio; *CI*, 95% confidence interval; *EDOS*, emergency department visits/observation stay; *M*, models 1–3; *N*, number; *RAI*, risk analysis index; *Ref*, reference value

30-day EDOS and readmission defined as 30 days from date of discharge from index hospitalization

EDOS and readmissions were evaluated independently. Membership in one group does not exclude a case from membership in the other. P values in bold indicate significance at the *p* < .05 level

#### Cost Analyses

### Decreased Index Hospitalization Variable Costs Among Patients with a 30-Day EDOS or Readmission

The %change of variable costs was higher for open abdomen versus laparoscopic cases (Supplemental Table 4 M1–M3). Medicaid/uninsured patients (12.1%, Supplemental Table 4 M1) had higher %changes in variable costs versus private but were similar after adjusting for urgent/emergent cases while Medicare patients had 11.2% higher costs versus private (Supplemental Table 4 M3). Having a stoma (32.0–55.3%), experiencing any complication (66.1–67.6%) and urgent/emergent cases (28.7%) increased index hospitalization costs. Patients with an EDOS and/or readmission versus those without had decreased (− 9.1 to − 10.8%) index hospitalization costs (Supplemental Table 4 M2 and M3).

### Decreased Index Hospitalization LOS Among Patients with a 30-Day EDOS or Readmission

The %change of LOS was higher for open abdomen versus laparoscopic cases and higher for patients with a stoma (Supplemental Table 5 M1–M3). After adjusting for complications and urgent/emergent cases, patients with a readmission and/or EDOS had lower relative difference LOS, than those without (Supplemental Table 5 M2 and M3). Medicaid/uninsured patients (16.9%, Supplemental Table 5 M1) had higher %changes in LOS versus private until adjusting for any complication and urgent/emergent cases (Supplemental Table 5 M3).

### Similar First EDOS and Readmission Costs between Private, Medicare, and Medicaid/Uninsured patients

The %change of the first EDOS and first readmission variable costs was similar for Medicare and Medicaid/uninsured versus private (Supplemental Table 6). The %change of the first readmission variable costs increased by 82.3% and 35.9% for cases experiencing any complication or were urgent/emergent, respectively.

### Increased Numbers of 30-Day EDOS and Readmissions in Medicaid/Uninsured Patients

Both Medicare and Medicaid/uninsured had increased rates of patients with EDOS, readmissions, and with both EDOS and readmission versus private. Medicaid/uninsured patients had the highest returns/case in each category (EDOS 0.22, readmissions 0.27, both 0.49; Table 4). Medicaid/uninsured patients had more successive EDOS and readmissions versus private. Multiple Medicaid/uninsured cases had between three and six EDOS within 30 days of discharge, as well as one case having four readmissions; none of the private or Medicare cases exhibited this increased frequency of multiple encounters.

**Table 4:** Increased numbers of 30-day^a^ EDOS and readmissions in Medicaid/uninsured patients

Outcomes		Private	Medicare	Medicaid/uninsured
All cases		245	195	590
No returns, *N* (%)		203 (82.9)	147 (75.4)	380 (64.4)
EDOS/readmissions, *N* (%)		42 (17.1)	48 (24.6)	210 (35.6)
Cumulative EDOS + readmissions/case		0.216	0.262	0.493
Cumulative EDOS/case		0.053	0.077	0.220
Cumulative readmissions/case		0.163	0.185	0.273
EDOS	Readmissions			
0	1	29	31	94
0	2	3	1	15
0	3			2
0	4			1
1	0	5	10	55
1	1	1		15
1	2	1	1	4
2	0	2	1	17
2	1		1	2
2	2	1		
3	0			2
3	1			2
** …**	** …**	**…**	**…**	**…**
6	0			1

*EDOS*, emergency department visits/observation stays; *N*, number ^a^30-day EDOS and readmission defined as 30 days from date of discharge from index hospitalization

### Increased Cumulative Variable Costs Among Patients with a 30-Day EDOS or Readmission

Both Medicare (17.0%) and Medicaid/uninsured patients (10.8%) had higher %changes in cumulative costs versus private (Table 5 M1), but only Medicaid/uninsured was similar to private after adjusting for urgent/emergent cases (Table 5 M3). Patients with a 30-day readmission and/or EDOS had higher %change (34.5%, Table 5 M1) than those without either even after adjusting for complications (14.8%, Table 5 M2) and urgent/emergent cases (16.9%, Table 5 M3). Stoma (31.3%), any complication (73.8%), and urgent/emergent cases (28.5%) increased cumulative hospitalization costs (Table 5 M3).

**Table 5:** Cumulative variable costs of index hospitalization plus all 30-day EDOS and readmissions nested models (M1–M3) adjusted for frailty, open abdominal procedures, insurance type, EDOS/readmissions, stoma status, any complication, and urgent/emergent cases

	Log(cumulative costs) M1				Log(cumulative costs) M2				Log(cumulative costs) M3			
Predictors	%	Est	CI	*p* value	%	Est	CI	*p* value	%	Est	CI	*p* value
Intercept		8.96	8.86–9.07	**< 0.001**		8.90	8.81–9.00	**< 0.001**		8.86	8.76–8.95	**< 0.001**
RAI (Ref = normal 21–29)												
Robust	− 8.28	− 0.09	− 0.17–0.00	**0.039**	− 7.08	− 0.07	− 0.15–0.00	0.054	− 5.43	− 0.06	− 0.13–0.02	0.133
Frail	6.73	0.07	− 0.06–0.20	0.326	3.74	0.04	− 0.08–0.15	0.537	2.79	0.03	− 0.09–0.14	0.635
Very frail	30.70	0.27	0.02–0.52	**0.038**	22.48	0.20	− 0.02–0.43	0.080	18.23	0.17	− 0.05–0.39	0.138
Open	28.25	0.25	0.18–0.32	**< 0.001**	15.69	0.15	0.08–0.21	**< 0.001**	10.59	0.10	0.03–0.17	**0.003**
Insurance (Ref = private)												
Medicare	16.96	0.16	0.04–0.27	**0.006**	12.18	0.11	0.01–0.22	**0.026**	12.05	0.11	0.02–0.21	**0.023**
Medicaid/uninsured	10.82	0.10	0.02–0.19	**0.016**	9.12	0.09	0.01–0.16	**0.023**	4.88	0.05	− 0.03–0.12	0.206
EDOS/readmissions	34.49	0.30	0.22–0.37	**< 0.001**	14.75	0.14	0.07–0.21	**< 0.001**	16.87	0.16	0.09–0.23	**< 0.001**
Stoma	56.09	0.45	0.37–0.52	**< 0.001**	36.71	0.31	0.24–0.38	**< 0.001**	31.26	0.27	0.20–0.34	**< 0.001**
Any complication					75.43	0.56	0.49–0.63	**< 0.001**	73.82	0.55	0.48–0.62	**< 0.001**
Urgent/emergent (Ref = elective)									28.53	0.25	0.19–0.32	**< 0.001**

*%*, %change; *CI*, 95% confidence interval; *EDOS*, emergency department visits/observation stays; *Est*, estimate; *Open*, open abdominal procedures (reference laparoscopic); *RAI*, risk analysis index; *Ref*, reference value

30-day EDOS and readmission defined as 30 days from date of discharge from index hospitalization

Note: %change is calculated with marginal change of Log(outcome) for one unit of each variable change ($e$^(intercept + estimated coefficients)^ − $e$ ^intercept^)/$e$ ^intercept^ *100, which is equal to ($e$ ^estimated coefficients^ − 1)*100. P values in bold indicate significance at the *p* < .05 level

### Increased Cumulative Return Costs Among Medicaid/Uninsured Patients

Medicaid/uninsured patients (138.7 to 181.4%) had increased %change in cumulative return costs versus private, even after adjusting for complications and urgent/emergent cases (Table 6, M1-M3).

**Table 6:** Cumulative return costs of all 30-day^a^ EDOS and readmissions using 3 nested models (M1–M3) adjusted for frailty, open abdominal procedures, insurance type, stoma status, any complication, and urgent/emergent cases

	Log(return costs) M1				Log(return costs) M2				Log(return costs) M3			
Predictors	%	Est	CI	*p* value	%	Est	CI	*p* value	%	Est	CI	*p* value
Intercept		0.44	− 0.21–1.08	0.185		0.16	− 0.46–0.78	0.617		0.24	− 0.39–0.86	0.458
RAI (Ref = normal 21–29)												
Robust	10.47	0.10	− 0.41–0.61	0.701	15.92	0.15	− 0.34–0.63	0.551	12.43	0.12	− 0.37–0.60	0.636
Frail	25.80	0.23	− 0.56–1.02	0.571	9.94	0.09	− 0.66–0.85	0.806	11.62	0.11	− 0.65–0.87	0.776
Very frail	691.6	2.07	0.53–3.61	**0.009**	425.5	1.66	0.19–3.13	**0.027**	454.8	1.71	0.24–3.18	**0.022**
Open	85.42	0.62	0.17–1.06	**0.006**	16.96	0.16	− 0.28–0.59	0.478	26.20	0.23	− 0.21–0.67	0.299
Insurance (Ref = private)												
Medicare	42.36	0.35	− 0.33–1.04	0.312	16.64	0.15	− 0.50–0.81	0.645	16.77	0.16	− 0.50–0.81	0.642
Medicaid/uninsured	181.4	1.03	0.53–1.54	**< 0.001**	138.7	0.87	0.39–1.35	**< 0.001**	154.2	0.93	0.45–1.42	**< 0.001**
Stoma	280.6	1.34	0.89–1.79	**< 0.001**	102.7	0.71	0.26–1.15	**0.002**	116.7	0.77	0.32–1.22	**< 0.001**
Any complication (Ref = none)					859.3	2.26	1.82–2.70	**< 0.001**	866.3	2.27	1.83–2.71	**< 0.001**
Urgent/emergent (Ref = elective)									− 34.70	− 0.43	− 0.85– − 0.00	**0.050**

%, %change; *CI*, 95% confidence interval; *EDOS*, emergency department visits/observation stays; *Est*, estimate; *Open*, open abdominal procedures (reference laparoscopic); *RAI*, risk analysis index; *Ref*, reference value. P values in bold indicate significance at the *p* < .05 level

^a^30-day EDOS and readmission defined as 30 days from date of discharge from index hospitalization

### Increased Mean and Median Cumulative Variable Costs Among Medicare and Medicaid/Uninsured Patients

Descriptive statistics for mean and median dollars for the cumulative variable costs of the index hospitalization and all 30-day EDOS and readmissions were reported due to the right skewed variable costs. Mean and median variable costs increased with open abdomen and urgent/emergent cases versus laparoscopic and elective cases, respectively in all three insurance groups (Table 7). Medicare, Medicaid/uninsured, and private mean variable costs were highest, intermediate and lowest, respectively, for all cases and each of the subgroups.

**Table 7:** Mean and median cumulative index hospitalization plus all EDOS and readmission variable costs ($) by insurance type for all cases and stratified by procedure type and case status

Means and medians	Overall	Private	Medicare	Medicaid/uninsured	*p* value^*a*^
All cases, number (%)^a^	1030	245 (23.8)	195 (18.9)	590 (57.3)	**< 0.001**
Mean (SD)	16,487 (17,708)	13,391 (17,706)	18,698 (17,916)	17,043 (17,501)	
Q1	7655	6723	8136	7974	
Median	11,013	9,274	11,722	11,755	
Q3	17.597	13,608	22,697	18,027	
Laparoscopic, number (%)	425 (41.3)	115 (46.9)	86 (44.1)	224 (38.0)	**< 0.001**
Mean (SD)	11,193 (9303)	9,687 (8,553)	13,035 (15,340)	11,258 (5915)	
Q1	6722	6461	6533	7015	
Median	8811	7552	8866	9596	
Q3	12,316	9788	12,627	13,236	
Open abdomen, number (%)	605 (58.7)	130 (53.1)	109 (55.9)	366 (62.0)	**< 0.001**
Mean (SD)	20,207 (20,973)	16,668 (22,479)	23,166 (18,593)	20,583 (20,971)	
Q1	9,116	8,486	10,495	8,999	
Median	13,565	11,329	17,983	13,690	
Q3	22,000	16,143	29,008	21,901	
Elective, number (%)	608 (59.0)	175 (71.4)	124 (63.6)	309 (52.4)	**0.019**
Mean (SD)	12,852 (12,487)	11,061 (9,120)	14,593 (15,571)	13,168 (12,662)	
Q1	6960	6636	7355	7011	
Median	9268	8567	9850	9318	
Q3	13,591	12,074	14,727	14,002	
Urgent/emergent, number (%)	422 (41.0)	70 (28.6)	71 (36.4)	281 (47.6)	**< 0.001**
Mean (SD)	21,725 (22,249)	19,215 (29,167)	25,867 (19,534)	21,303 (20,813)	
Q1	10,096	8,050	12,743	10,318	
Median	14,760	11,175	19,911	14,759	
Q3	23,902	16,496	31,508	21,924	

*SD*, standard deviation; *Q1*, first quartile; *Q3*, third quartile. P values in bold indicate significance at the *p* < .05 level

^a^Kruskal–Wallis test used for *p* values due to highly skewed cost data

## Discussion

This study comprehensively assessed the association of insurance type with EDOS, readmissions, and cumulative variable costs across CRS at an academic, SNH serving a diverse range of SES patients. Increased odds of complications, index hospitalization, return, and cumulative costs present in Medicaid/uninsured patients were driven by higher rates and increased odds of urgent/emergent cases. Urgent/emergent cases were associated with higher odds of having a stoma and open abdominal procedures; further contributing to the increased odds of complications, EDOS, and readmissions. However, the three insurance groups had similar odds of complications in the stoma subgroup, suggesting that the need to create a stoma was the driving factor in outcomes, rather than insurance type.

Increased index hospitalization and cumulative variable costs among Medicaid/uninsured insurance patients were similar to private after adjusting for urgent/emergent cases, while Medicare patients had higher %change even after adjusting for case status. Demographics for age and frailty scores were similar between private and Medicaid/uninsured patients, compared to Medicare patients whom were older with higher frailty scores potentially contributing to the increased costs. Open abdominal procedures, presence of a stoma, complications, and urgent/emergent cases were associated with increased index hospitalization and cumulative costs. The variation in index hospitalization costs by insurance type was consistent with prior studies^49^ including complications having the highest associated costs.^50,51^ Our study demonstrated that Medicaid/uninsured patients undergo more urgent/emergent surgeries, increasing the odds of open abdominal surgery, which was associated with more complications and higher costs versus private. Medicare and Medicaid/uninsured patients cost more and reimburse less than patients with private insurance.^52,53^

This study is one of the first to evaluate the return and cumulative costs of multiple EDOS and readmissions. While the 1^st^ EDOS and readmission costs were similar across insurance type, return costs were 154% higher for Medicaid/uninsured patients due to the increased frequency of EDOS and readmission encounters even after adjusting for urgent/emergent cases. Medicaid/uninsured patients had higher odds of readmissions versus private after adjusting for urgent/emergent cases but not after adjusting for complications. Our Medicaid/uninsured group included patients with dual enrollment in Medicare/Medicaid. The use of readmissions as a quality metric has serious implications. Patient factors,^54^ urgent/emergency surgeries,^55,56^ and complications after discharge^57^ are major factors driving readmissions and return visits. Though SNH have higher readmission rates,^25^ studies show SNH provide similar quality care to low-burden hospitals.^58–61^ However, multiple studies demonstrate that SNH are disproportionately penalized by HRRP,^22,23,62^ worsening disparities between SNH that largely care for vulnerable populations.^17,23^ The increased index hospitalization, cumulative, and return costs and penalties for worse outcomes^22,23,62^ further strain resources at SNH. Including risk adjustment for urgent and emergent cases and could improve the accuracy of quality metrics. A possible solution to decreasing costs from urgent/emergent surgeries is improving outpatient and preventive care for Medicaid/Uninsured patients. Furthermore, the use of readmissions as a quality metric has been criticized as readmissions are heavily influenced by medical conditions and social factors that hospitals and clinicians cannot reasonably control.^63^ While the majority of procedures in our cohort are not covered by HRRP, we think it is important to study surgical readmissions to inform future decisions by the Centers for Medicare and Medicaid Services and private insurance companies regarding expanding or initiating readmissions policies.

Medicaid/uninsured patients had higher odds of EDOS versus private even after adjusting for complications. Studies suggest that many emergency department visits after CRS could be prevented.^64–66^ Our data suggests that Medicaid/uninsured patients are using the emergency department as substitute for outpatient care, further adding to costs.

### Limitations

This study used a retrospective cohort and does not establish causal relationships. NSQIP provides a representative sample of surgeries but does not include all procedures for a healthcare system. This study only examined the clinical outcomes and costs observed by a single institution. Outside hospital EDOS and readmissions were not included secondary to missing cost data and our focus was on the impact of Medicaid/uninsured patients on SNH costs. We used complications available in NSQIP; various surgeries have specific complications not included in these analyses.

## Conclusions

Increased rates and odds of urgent/emergent colorectal cases in Medicaid/uninsured populations drive increased odds of complications and higher index hospitalization and cumulative costs versus private patients suggesting lack of access to outpatient care. Return costs were were 154% higher for Medicaid/uninsured patients versus private due to the increased frequency of EDOS and readmission encounters. SNH care for higher cost populations while receiving lower reimbursements and are further penalized by current value-based programs such as HRRP. Increasing access to care for Medicaid/uninsured patients could reduce urgent/emergent surgeries resulting in fewer complications, EDOS/readmissions, and costs.

## Conflict of Interest

Dr. Schmidt reported receiving grants from the National Institutes of Health and Veterans Health Administration. Dr. Shireman reported receiving grants from the National Institutes of Health and Veterans Health Administration and salary support from Texas A&M University, South Texas Veterans Health Care System and the University of Texas Health San Antonio during the conduct of the study. No other disclosures were reported.

## Disclaimer

The funding sources had no role in the design and conduct of the study; collection, management, analysis, and interpretation of the data; preparation, review, or approval of the manuscript; and decision to submit the manuscript for publication. The opinions expressed here are those of the authors and do not necessarily reflect the position of the United States government.

## Supplementary Information

Below is the link to the electronic supplementary material.

Supplementary file1 (DOCX 305 KB)

## Data Availability

The data for this study is not available as it contains proprietary cost data and National Surgical Quality Improvement Program data.
