# Supplementary material for: Association of Cumulative Colorectal Surgery Hospital Costs, Readmissions, and Emergency Department/Observation Stays with Insurance Type
Source: J Gastrointest Surg. 2023 Jan 23;27(5):965–79. doi: 10.1007/s11605-022-05576-7 (PMC10133377; doi:10.1007/s11605-022-05576-7)
Supplement: Supplementary file 1 — Supplementary file1 (DOCX 305 KB) [file 11605_2022_5576_MOESM1_ESM.docx]

| **Supplemental Table 1. Principal Current Procedural Terminology (CPT) Codes Used to Identify Colorectal Surgery Type**   \| **INCLUDED** \| \| \| **EXCLUDED** \| \| \| --- \| --- \| --- \| --- \| --- \| \| **Laparoscopic Procedures** \| **Open Abdominal Procedures^a^** \| **Stoma Absence or Presence** \| **Perineal Procedures** \| **Transsacral Procedures** \| \| 44204 44205 44206 44207 44208 44210 44211 44212 45395 45397 45402 \| 44140 44141 44143 44144 44145 44146 44147 44150 44151 44155 44156 44157 44158 44160 45110 45111 45112 45113 45114 45119 45120 45121 45126 45135 45550 \| ***Colorectal procedures with a primary anastomosis***  44140, 44145, 44147, 44160, 44204, 44205, 44207, 45112, 45114, 45116, 45120, 45121, 45130, 45135, 45402, 45550  ***Colorectal procedures not requiring an anastomosis or stoma***  45123, 45160  ***Colorectal procedures possibly having a stoma^b^***  44130, 44150, 44157, 44158, 44210, 44389, 45113, 45119, 45126, 45397  ***Colorectal procedures with a stoma***  44141, 44143, 44144, 44146, 44151, 44155, 44156, 44206, 44208, 44211, 44212, 45110, 45111, 45395  ***Other CPT codes used to identify a stoma^c^***  44125, 44187, 44188, 44310, 44312, 44314, 44316, 44320, 44322, 44340, 44345, 44346, 44380, 44382, 44381, 44384, 44385, 44386, 44388, 44389, 44390, 44394, 44391, 44392, 44401, 44402, 44403, 44404, 44405, 44406, 44407, 44408, 44605, 45136, 45563, 45805, 45825 \| 45123  45130 \| 45116  45160 \| |
| --- | --- | --- | --- | --- | --- | --- | --- | --- | --- | --- | --- | --- | --- | --- | --- |
| ^a^Includes combined open abdominal and perineal procedures  ^b^Chart review used to determine presence of a stoma  ^c^Presence of a stoma if CPT code used during index hospitalization |

**Supplemental Table 2. National Surgical Quality Improvement Program (NSQIP) Variables used for Any Complication**

| NSQIP Variable Name | NSQIP Definition |
| --- | --- |
| REINTUB | Intubation intraoperatively or within 30 days after surgery |
| PULEMBOL | New diagnosis of a pulmonary embolism within 30 days after surgery |
| FAILWEAN | Requirement of a ventilator for more than 48 cumulative hours within 30 days after surgery |
| OPRENAFL | Renal failure requiring dialysis within 30 days after surgery |
| CNSCVA | Cerebral Vascular Accident or stroke with motor, sensory, or cognitive dysfunction for 24 or more hours within 30 days after surgery |
| CDARREST | Chaotic or absent cardiac rhythm requiring CPR within 30 days after surgery |
| CDMI | Acute myocardial infarction which occurred intraoperatively or within 30 days after surgery |
| OTHSESHOCK | Sepsis associated with organ and/or circulatory dysfunction within 30 days after surgery |
| SUPINFEC | Superficial incisional surgical site infection that occurs within 30 days after surgery |
| WNDINFD | Deep incision surgical site infection that occurs within 30 days after surgery |
| ORGSPCSSI | Organ/Space surgical site infection that occurs within 30 days after surgery |
| DEHIS | Wound separation that compromises integrity of closure, occurring within 30 days after surgery |
| OUPNEUMO | Pneumonia that occurs within 30 days after surgery |
| RENAINSF | Reduced kidney capacity (without requirement for dialysis) within 30 days after surgery |
| URNINFEC | Infection in the kidneys, ureters, bladder, or urethra, occurring within 30 days after surgery |
| OTHBLEED | Use of one or more units of packed or whole red blood cells intraoperatively or within 72 hours after surgery |
| OTHDVT | New diagnosis of blood clot or thrombus within the venous system, occurring within 30 days after surgery |
| OTHCDIFF | C. difficile colitis within 30 days after surgery |
| OTHSYSEP | Sepsis within 30 days after surgery |
| REOPERATION1 | Unplanned return to the operating room for a surgical procedure, for any reason, within 30 days after surgery |

**Supplemental Table 3. Distribution of Medicaid/Uninsured Insurance Group**

| **Medicaid/Uninsured Group Total** | **590** |
| --- | --- |
| County Indigent Programs, No. (%) | 272 (46.1) |
| Charity care, No. (%) | 8 (1.4) |
| Medicare/Medicaid Dual Enrollment, No. (%) | 64 (10.8) |
| Medicaid, No. (%) | 150 (25.4) |
| Self-pay <1% charges collected, No. (%) | 96 (16.3) |

Case from patients that had dual enrollment in Medicare and Medicaid were assigned to the Medicaid/Uninsured group

**
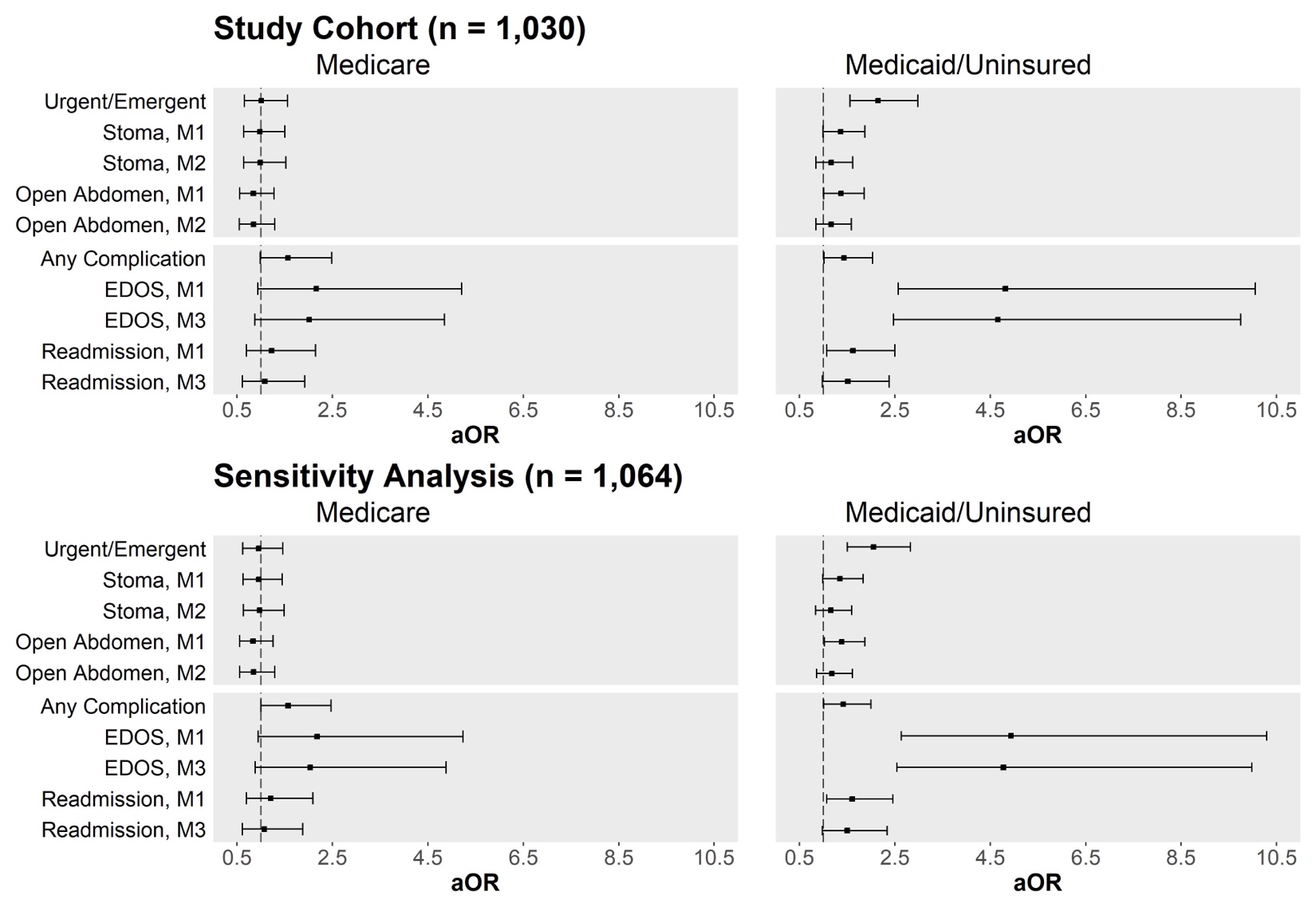
**

**Supplementary Figure 1. Sensitivity Analysis for Clinical Outcomes– Forest Plot Comparing Study Cohort to Cohort with Cases Added (n = 34) for Patient Mortality Resulting in No or Reduced Chances of Subsequent 30-day EDOS and Readmissions**

Forest plots of **Tables 2-3** (top) for Medicare and Medicaid/Uninsured compared to Private insurance patients with a sensitivity analysis adding cases excluded due to 1) death during the index hospitalization, 2) discharge to another acute care hospital, 3) discharge against medical advice, 4) death within 30 days of discharge when discharged to Hospice or Home on Hospice, and 5) death within 30 days of discharge without a 30-day EDOS or readmission (bottom).

Clinical Outcomes models from **Table 2** of Urgent/Emergent Cases, Stoma M1 and Open Abdomen M1 were adjusted for RAI. Stoma M2 and Open Abdomen M2 were also adjusted for Urgent/Emergent Cases in addition to RAI.

Clinical outcome models from **Table 3** of Any Complication, EDOS M1 and Readmission M1 were adjusted for RAI, Open Abdomen, Urgent/Emergent cases and Stoma status. EDOS M3 and Readmission M3 were also adjusted for Any Complication in addition to the variables listed for the M1 models.

Abbreviations: aOR, Adjusted Odds Ratio; EDOS; Emergency Department visits/Observation Stays; RAI, Risk Analysis Index

30-day EDOS and Readmission defined as 30 days from date of discharge from index hospitalization

**Supplemental Table 4. Index Hospitalization Variable Costs using 3 Nested Models (M1-M3) Adjusted for Frailty, Open Abdominal Procedures, Insurance Type, EDOS/Readmissions, Stoma Status, Any Complication and Urgent/Emergent Cases**

|  | **Log(Index Variable Costs) M1** | | | | | | **Log(Index Variable Costs) M2** | | | | | | | **Log(Index Variable Costs) M3** | | | |  |
| --- | --- | --- | --- | --- | --- | --- | --- | --- | --- | --- | --- | --- | --- | --- | --- | --- | --- | --- |
| *Predictor* | *%* | *Est* | *CI* | | *p-value* | | | *%* | | *Est* | | *CI* | *p-value* | *%* | *Est* | *CI* | *p-value* | |
| Intercept |  | 8.96 | 8.85 – 9.06 | | **<.001** | | |  | | 8.90 | | 8.81 – 9.00 | **<.001** |  | 8.86 | 8.76 – 8.95 | **<.001** | |
| **RAI (Ref = Normal 21-29)** | | | |  | |  | | |  | |  | |  |  |  |  |  | |
| Robust | -8.08 | -0.08 | -0.17 – -0.00 | | **.048** | | | -6.88 | | -0.07 | | -0.15 – 0.01 | .068 | -5.21 | -0.05 | -0.13 – 0.02 | .160 | |
| Frail | 9.18 | 0.09 | -0.04 – 0.22 | | .187 | | | 6.37 | | 0.06 | | -0.06 – 0.18 | .312 | 5.39 | 0.05 | -0.06 – 0.17 | .377 | |
| Very Frail | 30.70 | 0.27 | 0.01 – 0.52 | | **.039** | | | 23.13 | | 0.21 | | -0.02 – 0.44 | .080 | 18.83 | 0.17 | -0.05 – 0.40 | .136 | |
| **Open Abdomen** | 27.91 | 0.25 | 0.17 – 0.32 | | **<.001** | | | 16.35 | | 0.15 | | 0.08 – 0.22 | **<.001** | 11.19 | 0.11 | 0.04 – 0.17 | **.002** | |
| **(Ref = Laparoscopic)** | | | | | | | | | |  | |  |  |  |  |  |  | |
| **Insurance (Ref = Private)** | | | |  | |  | | |  | |  | |  |  |  |  |  | |
| Medicare | 15.63 | 0.15 | 0.03 – 0.26 | | **.012** | | | 11.29 | | 0.11 | | 0.00 – 0.21 | **.043** | 11.16 | 0.11 | 0.01 – 0.21 | **.039** | |
| Medicaid/Uninsured | 12.06 | 0.11 | 0.03 – 0.20 | | **.008** | | | 10.49 | | 0.10 | | 0.02 – 0.18 | **.011** | 6.17 | 0.06 | -0.02 – 0.14 | .121 | |
| **EDOS/Readmissions** | 3.24 | 0.03 | -0.05 – 0.11 | | .419 | | | -10.8 | | -0.11 | | -0.19 – -0.04 | **.003** | -9.10 | -0.10 | -0.17 – -0.02 | **.010** | |
| **Stoma (Ref = None)** | 55.32 | 0.44 | 0.37 – 0.51 | | **<.001** | | | 37.50 | | 0.32 | | 0.25 – 0.39 | **<.001** | 31.99 | 0.28 | 0.21 – 0.35 | **<.001** | |
| **Any Complication (Ref = None)** | | | |  | | 67.61 | | | 0.52 | | 0.44 – 0.59 | | **<.001** | 66.06 | 0.51 | 0.44 – 0.58 | **<.001** | |
| **Urgent/Emergent (Ref = Elective)** | | | |  | |  | | |  | |  | |  | 28.72 | 0.25 | 0.29 – 0.32 | **<.001** | |

Abbreviations: CI, 95% Confidence Interval; EDOS; Emergency Department visits/Observation Stays; Est; Estimate; %, %change; RAI, Risk Analysis Index; Ref, Reference Value
30-day EDOS and Readmission defined as 30 days from date of discharge from index hospitalization
Note: %change is calculated with marginal change of Log(outcome) for one unit of each variable change

($e$^(intercept+estimated coefficients)^ -$e$^intercept^ )/$e$^intercept^ *100, which is equal to ($e$^estimated coefficients^ -1)*100

**Supplemental Table 5.** **Index Hospitalization Length of Stay Using 3 Nested Models (M1-M3) Adjusted for Frailty, Open Abdominal Procedures, Insurance Type, EDOS/Readmissions, Stoma Status, Any Complication and Urgent/Emergent Cases**

|  | **Log(Length of Stay) M1** | | | | | | **Log(Length of Stay) M2** | | | | **Log (Length of Stay) M3** | | | | |  |
| --- | --- | --- | --- | --- | --- | --- | --- | --- | --- | --- | --- | --- | --- | --- | --- | --- |
| *Predictor* | *%* | *Est* | | *CI* | | *p-value* | *%* | *Est* | *CI* | *p-value* | | *%* | *Est* | *CI* | *p-value* | |
| **Intercept** |  | 1.69 | | 1.57 – 1.80 | | **<.001** |  | 1.64 | 1.53 – 1.74 | **<.001** | |  | 1.55 | 1.45 – 1.65 | **<.001** | |
| **RAI (Ref = Normal 21-29)** | |  | |  | |  |  |  |  |  | |  |  |  |  | |
| Robust | -10.27 | -0.11 | | -0.20 – -0.02 | | **.017** | -9.19 | -0.10 | -0.18 – -0.01 | **.024** | | -6.30 | -0.07 | -0.14 – 0.01 | .101 | |
| Frail | 7.64 | 0.07 | | -0.07 – 0.21 | | .299 | 5.09 | 0.05 | -0.08 – 0.18 | .456 | | 3.39 | 0.03 | -0.09 – 0.15 | .590 | |
| Very Frail | 30.83 | 0.27 | | -0.00 – 0.54 | | .051 | 23.84 | 0.21 | -0.04 – 0.47 | .099 | | 16.31 | 0.15 | -0.09 – 0.39 | .210 | |
| **Open Abdomen (Ref = Laparoscopic)** | 42.26 | 0.35 | | 0.27 – 0.43 | | **<.001** | 30.37 | 0.27 | 0.19 – 0.34 | **<.001** | | 20.35 | 0.19 | 0.11 – 0.26 | **<.001** | |
| **Insurance (Ref = Private)** | |  | |  | |  |  |  |  |  | |  |  |  |  | |
| Medicare | 11.04 | 0.10 | | -0.02 – 0.22 | | .087 | 7.19 | 0.07 | -0.04 – 0.18 | .227 | | 6.98 | 0.07 | -0.04 – 0.17 | .207 | |
| Medicaid/Uninsured | 16.94 | 0.16 | | 0.07 – 0.25 | | **<.001** | 15.42 | 0.14 | 0.06 – 0.23 | **<.001** | | 7.59 | 0.07 | -0.01 – 0.15 | .069 | |
| **EDOS/Readmission** | 0.35 | 0.00 | | -0.08 – 0.09 | | .935 | -12.27 | -0.13 | -0.21 – -0.05 | **.002** | | -9.37 | -0.10 | -0.17 – -0.02 | **.010** | |
| **Stoma (Ref = None)** | 53.28 | 0.43 | | 0.35 – 0.51 | | **<.001** | 37.01 | 0.31 | 0.24 – 0.39 | **<.001** | | 27.48 | 0.24 | 0.17 – 0.32 | **<.001** | |
| **Any Complication (Ref = None)** | | |  | |  | | 60.93 | 0.48 | 0.40 – 0.55 | **<.001** | | 58.31 | 0.46 | 0.39 – 0.53 | **<.001** | |
| **Urgent/Emergent (Ref = Elective)** | | |  | |  | |  |  |  |  | | 56.08 | 0.45 | 0.38 – 0.51 | **<.001** | |

Abbreviations: CI, 95% Confidence Interval; EDOS; Emergency Department visits/Observation Stays; Est; Estimate; %, %change; RAI, Risk Analysis Index; Ref, Reference Value;
30-day EDOS and Readmission defined as 30 days from date of discharge from index hospitalization

**Supplemental Table 6.** **Variable Costs for 1st 30-day EDOS and 1st 30-day Readmission Adjusted for RAI, Open Abdominal Procedures, Insurance Type, Stoma Status, Any Complication and Urgent/Emergent Cases**

|  | **Log(1^st^ EDOS Variable Costs)** | | | | | | **Log(1^st^ Readmission Variable Costs)** | | | | | | | | | | | |  |
| --- | --- | --- | --- | --- | --- | --- | --- | --- | --- | --- | --- | --- | --- | --- | --- | --- | --- | --- | --- |
| *Predictor* | *%* | *Est* | | *CI* | | *p-value* | | | *%* | | | *Est* | | | *CI* | | | *p-value* | |
| **Intercept** |  | 4.80 | | 3.70 – 5.90 | | **<.001** | |  | | | 7.80 | | | 7.33 – 8.27 | | | **<.001** | |  |
| **RAI (Ref = Normal 21-29)** | | |  | |  | |  | | |  | | |  | | |  | | |  |
| Robust | 4.29 | 0.04 | | -0.57 – 0.65 | | .892 | | -25.62 | | | -0.30 | | | -0.60 – 0.01 | | | .058 | |  |
| Frail | -46.02 | -0.62 | | -1.58 – 0.35 | | .207 | | -19.77 | | | -0.22 | | | -0.64 – 0.20 | | | .299 | |  |
| Very Frail | 76.70 | 0.57 | | -1.11 – 2.25 | | .503 | | -18.27 | | | -0.20 | | | -0.80 – 0.39 | | | .504 | |  |
| **Open Abdomen**  **(Ref = Laparoscopic)** | 22.95 | 0.21 | | -0.35 – 0.77 | | .466 | | -2.32 | | | -0.02 | | | -0.31 – 0.26 | | | .871 | |  |
| **Insurance (Ref = Private)** | | |  | |  | |  | | |  | | |  | | |  | | |  |
| Medicare | 55.01 | 0.44 | | -0.69 – 1.57 | | .443 | | 0.51 | | | 0.01 | | | -0.42 – 0.43 | | | .981 | |  |
| Medicaid/Uninsured | -14.10 | -0.15 | | -1.06 – 0.75 | | .740 | | -1.96 | | | -0.02 | | | -0.35 – 0.31 | | | .905 | |  |
| **Stoma (Ref = None)** | 42.57 | 0.35 | | -0.16 – 0.87 | | .179 | | 11.18 | | | 0.11 | | | -0.16 – 0.37 | | | .434 | |  |
| **Any Complication**  **(Ref = None)** | 29.33 | 0.26 | | -0.27 – 0.79 | | .338 | | 82.31 | | | 0.60 | | | 0.32 – 0.89 | | | **<.001** | |  |
| **Urgent/Emergent**  **(Ref = Elective)** | -16.27 | -0.18 | | -0.71 – 0.35 | | .507 | | 35.85 | | | 0.31 | | | 0.05 – 0.56 | | | **.017** | |  |

Abbreviations: CI, 95% Confidence Interval; EDOS; Emergency Department visits/Observation Stays; Est; Estimate; %, %change; RAI, Risk Analysis Index; Ref, Reference Value;
30-day EDOS and Readmission defined as 30 days from date of discharge from index hospitalization

Readmissions and EDOS were evaluated independently; membership in one group does not exclude a case from membership in the other
